# Roles of anthropogenic forcings in the observed trend of decreasing late-summer precipitation over the East Asian transitional climate zone

**DOI:** 10.1038/s41598-021-84470-9

**Published:** 2021-03-02

**Authors:** Wei Zhao, Wen Chen, Shangfeng Chen, Hainan Gong, Tianjiao Ma

**Affiliations:** 1 National Meteorological Center of China Meteorological Administration, Beijing, China; 2 Center for Monsoon System Research, Institute of Atmospheric Physics, Chinese Academy of Sciences, Beijing, China; 3 College of Earth and Planetary Sciences, University of Chinese Academy of Sciences, Beijing, China

**Keywords:** Atmospheric dynamics, Attribution

## Abstract

Observations indicate that late-summer precipitation over the East Asian transitional climate zone (TCZ) showed a pronounced decreasing trend during 1951–2005. This study examines the relative contributions of anthropogenic [including anthropogenic aerosol (AA) and greenhouse gas (GHG)] and natural forcings to the drying trend of the East Asian TCZ based on simulations from CMIP5. The results indicate that AA forcing plays a dominant role in contributing to the drying trend of the TCZ. AA forcing weakens the East Asian summer monsoon via reducing the land-sea thermal contrast, which induces strong low-level northerly anomalies over eastern China, suppresses water vapor transport from southern oceans and results in drier conditions over the TCZ. In contrast, GHG forcing leads to a wetting trend in the TCZ by inducing southerly wind anomalies, thereby offsetting the effect of the AA forcing. Natural forcing has a weak impact on the drying trend of the TCZ due to the weak response of atmospheric anomalies.

## Introduction

In the context of global warming, the impacts of human-induced climate change, including more extreme weather events, sea-level rise, and sea ice melting, are threatening human society on both continental and regional scales^1^. The transitional climate zone (TCZ) in East Asia, which is located between the arid and humid regions, is found to be particularly susceptible to human activities^2–6^. Observational evidence shows that precipitation over the northern part of East Asia has experienced a pronounced decreasing trend over several recent decades^7–10^. Droughts and water deficiencies caused by decreasing precipitation trends have severely jeopardized local agriculture, ecosystems and socioeconomic development^2,8,11^. For instance, provinces in northern China encountered an extreme consecutive drought event in summer of 1997, which damaged the croup area more than 13 thousand square kilometers and induced severe locust disaster^12,13^. Previous studies have demonstrated that the decreasing precipitation in northern China during recent decades is probably linked to the interdecadal shift in the extratropical air–sea coupling mode (e.g., Pacific decadal oscillation (PDO) and Atlantic multidecadal oscillation (AMO))^9,10^. Specifically, Qian and Zhou^9^ noted that North China is dominated by a strong anomalous anticyclone (cyclone) in the lower troposphere during the positive (negative) phase of PDO, which contributes to a drier (wetter) conditions there.

Recent studies have indicated that in addition to the effects of internal climate variability, external forcings have also had a significant impact on the recent drying trend in northern China^14–18^. For example, based on the air–sea coupling model simulations, Wang et al.^17^ demonstrated that surface cooling over the Yangzi River Valley caused by increasing anthropogenic aerosol (AA) levels and associated strong convection reduces the thermal contrast in the East Asia region, which weakens the East Asia summer monsoon and leads to more frequent drought events in northern China. Based on the simulations from the Coupled Model Intercomparison Project phase 5 (CMIP5), Zhao et al.^14^ suggested that greenhouse gas (GHG) forcing is the dominant factor for the wetting trend in the arid and semiarid regions in China since the 1970s, whereas the AA forcing has produced a drying trend in the humid and semihumid regions.

The above studies primarily examined the role of external or natural forcing in the regional precipitation trend over the East Asian monsoon region separately. In addition, as a transitional area with sharp biome gradients, the TCZ in East Asia has a more fragile ecosystem and has suffered from more frequent drought events in recent decades^19,20^. Until now, there has been a gap in our knowledge regarding the relative contributions of different external forcings to the precipitation trend over the East Asian TCZ in recent decades. In this study, we aim to address this issue based on observations and multimodel simulations from CMIP5. The results will help improve the understanding of drought mechanisms over the East Asian TCZ, which is of great societal concern.

## Data and methods

The data utilized in this study include (1) high-resolution monthly precipitation and surface air temperature (SAT) data from the gridded Climatic Research Unit (CRU) Time-series (TS) data version 4.01 provided by the University of East Anglia, which include observations from 4000 stations on land around the world, cover the period of 1901–2016^21^, and have a horizontal longitude-latitude grid resolution of 0.5° × 0.5°; (2) monthly precipitation data from the Global Precipitation Climatology Centre (GPCC) with a horizontal resolution of 1° × 1°, spanning from 1901 to 2010^22^; (3) monthly atmospheric circulation variables including the 850-hPa horizontal winds and sea-level pressure (SLP) extracted from the National Centers for Environmental Prediction–National Center for Atmospheric Research (NCEP–NCAR)^23^, with a horizontal resolution of 2.5° × 2.5° and covering the period from 1948 to the present; and (4) monthly sea surface temperature (SST) data from the NOAA Extended Reconstructed SST version 3b dataset (ERSSTv3b), which has a horizontal resolution of 2° × 2° and is available from 1854 to the present^24^.

The simulation data from outputs of CMIP5 models’ experiments (i.e., historical, historical GHG, and historical natural experiments) are used to analyze the contributions of individual external and natural forcings. For details of the CMIP5 models, refer to Table S1 in the supporting information. Here, historical simulations are forced by both natural (e.g., solar variability, ozone, volcanic aerosols, etc.) and anthropogenic (e.g., GHG and AA) forcings^25^. The historical GHG (referred to as GHG forcing) and historical natural (referred to as natural forcing) experiments are similar to the historical run (the multimodel ensemble mean (MME) of historical runs is referred to as all forcing), except that the external forcings are well-mixed GHG changes or natural variations. Following Taylor et al.^25^, the response to anthropogenic forcing is regarded as the difference between the all forcing and natural forcing runs. The response of AA forcing is defined as the difference between the anthropogenic forcing and GHG forcing runs^25^. Both observational and model data are interpreted bilinearly into a common horizontal resolution of 0.5° × 0.5° to calculate the MME.

Since the CMIP5 outputs do not cover the data after 2005, this study focused on the period of 1951–2005 when all observations and simulations are available. The linear trend is evaluated by a least-squares regression analysis. The two-tailed Student’s *t* test is applied to evaluate the statistical significance of the linear trend.

## Results

**Figure 1:**
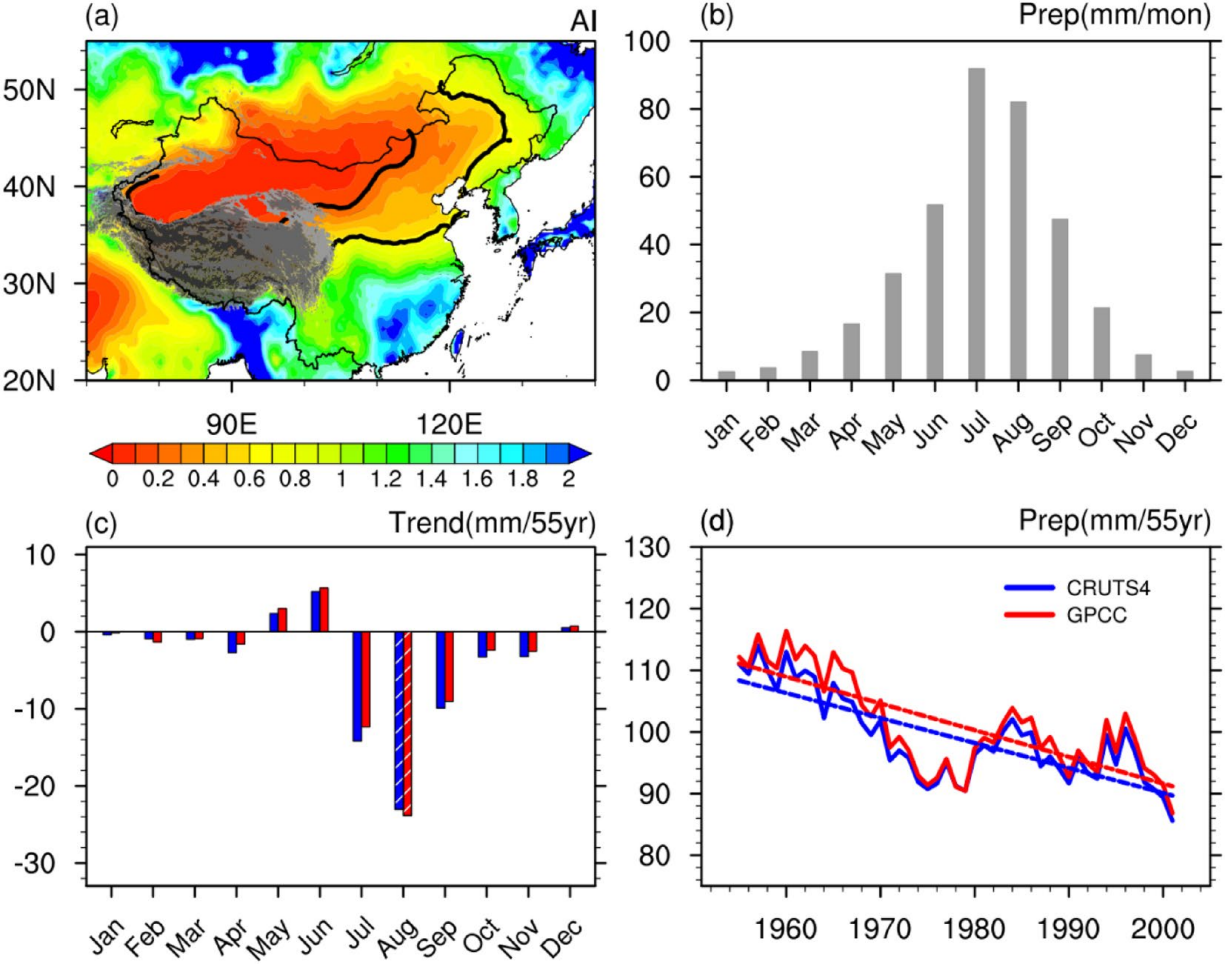
(**a**) Spatial distribution of climatological aridity index during 1951–2005. Gray shadings denote the Tibetan Plateau. (**b**) Climatological monthly precipitation averaged over the TCZ of East Asia during 1951–2005. (**c**) Linear trend of monthly precipitation (unit: mm month^−1^ 55 yr^−1^) averaged over the TCZ during 1951–2005 derived from CRU TS version 4.01^21^ (URL: http://crudata.uea.ac.uk/cru/data/hrg/cru_ts_4.01/) (blue bars) and GPCC V7^22^ (URL: https://psl.noaa.gov/data/gridded/data.gpcc.html) (red bars). Bars with slashes indicate linear trends significant at the 90% confidence level. (**d**) Nine-year running mean (solid lines) and linear trend (dashed lines) of the August precipitation over the TCZ during 1951–2005 derived from CRU TS version 4.01^21^ (blue lines) and GPCC V7^22^ (red lines). This Figure is created by the NCAR Command Language (version 6.4.0 & URL: http://www.ncl.ucar.edu/Download)^27^.

Figure 1a shows the spatial distribution of the climatological aridity index during 1951–2005 and the geographic location of the TCZ in East Asia (indicated by the areas between the two black solid curves). This study identifies the TCZ following the definition of Wang et al.^26^. According to Wang et al.^26^, the TCZ in East Asia is regarded as a combination of semiarid and dry subhumid areas, where the aridity index ranges from 0.2 to 0.65 (Fig. 1a). Here, the aridity index is defined as the ratio of annual precipitation and annual potential evaporation, which classifies the climate types globally^26^.

The climatological monthly precipitation averaged over the TCZ during 1951–2005 is presented in Fig. 1b. The greatest precipitation occurs in July (Fig. 1b), and the precipitation in August over the TCZ is comparable to that in July. As indicated by Zhao et al.^28^, July and August correspond to the main rainy season in the TCZ in East Asia. Figure 1c exhibits long-term linear trends of monthly precipitation over the TCZ. The results derived from CRU and GPCC are highly consistent (Fig. 1c). Except for May, June and December, all other months showed decreasing precipitation trends. Moreover, a statistically significant trend can only be detected in August (i.e., late summer), significant at the 90% confidence level. The amplitudes of the negative trends in July and September are approximately half that in August but are not significant (Fig. 1). Therefore, in the following, we focus on investigating the trend of August precipitation over the TCZ. The reasons that why significant decreasing trend of TCZ precipitation is found only in August may be complicated and needs to be further investigated in future.

Figure 1d displays the 9-year running mean of the August precipitation over the TCZ. The decreasing rates of precipitation obtained from CRU and GPCC are similar. It is noted that TCZ precipitation in August has been consistently decreasing since the early 1950s, with only small rebounds in the early 1980s and mid-1990s (Fig. 1d). It implies that the long-term decreasing trend of August precipitation over the TCZ since the early 1950s may be mainly attributed to external forcings rather than the internal climate variability. This conclusion is further confirmed below. The significant declining trend of TCZ precipitation in August can also be observed during the period of 1951–2012. This suggests that results obtained in this study are insensitive to slight changes of the time period.

**Figure 2:**
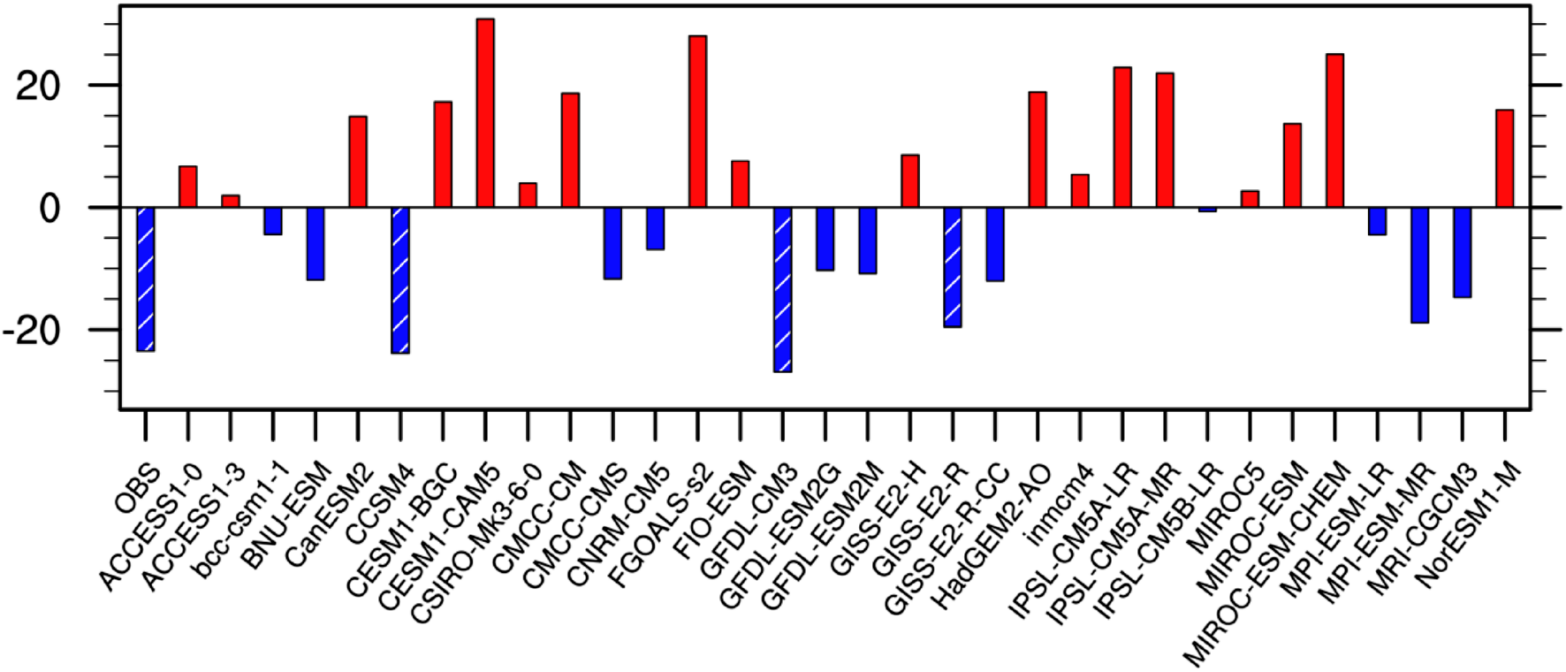
Linear trend of precipitation (unit: mm month^−1^ 55 yr^−1^) in August averaged over the TCZ during 1951–2005 in the observation and 32 CMIP5 models. The observation trend is calculated based on the average precipitation derived from CRU TS version 4.01^21^ (URL: http://crudata.uea.ac.uk/cru/data/hrg/cru_ts_4.01/) and GPCC V7^22^ (URL: https://psl.noaa.gov/data/gridded/data.gpcc.html). Bars with slashes indicate linear trends exceeding the 90% confidence level. This Figure is created by the NCAR Command Language (version 6.4.0 & URL: http://www.ncl.ucar.edu/Download)^27^.

**Figure 3:**
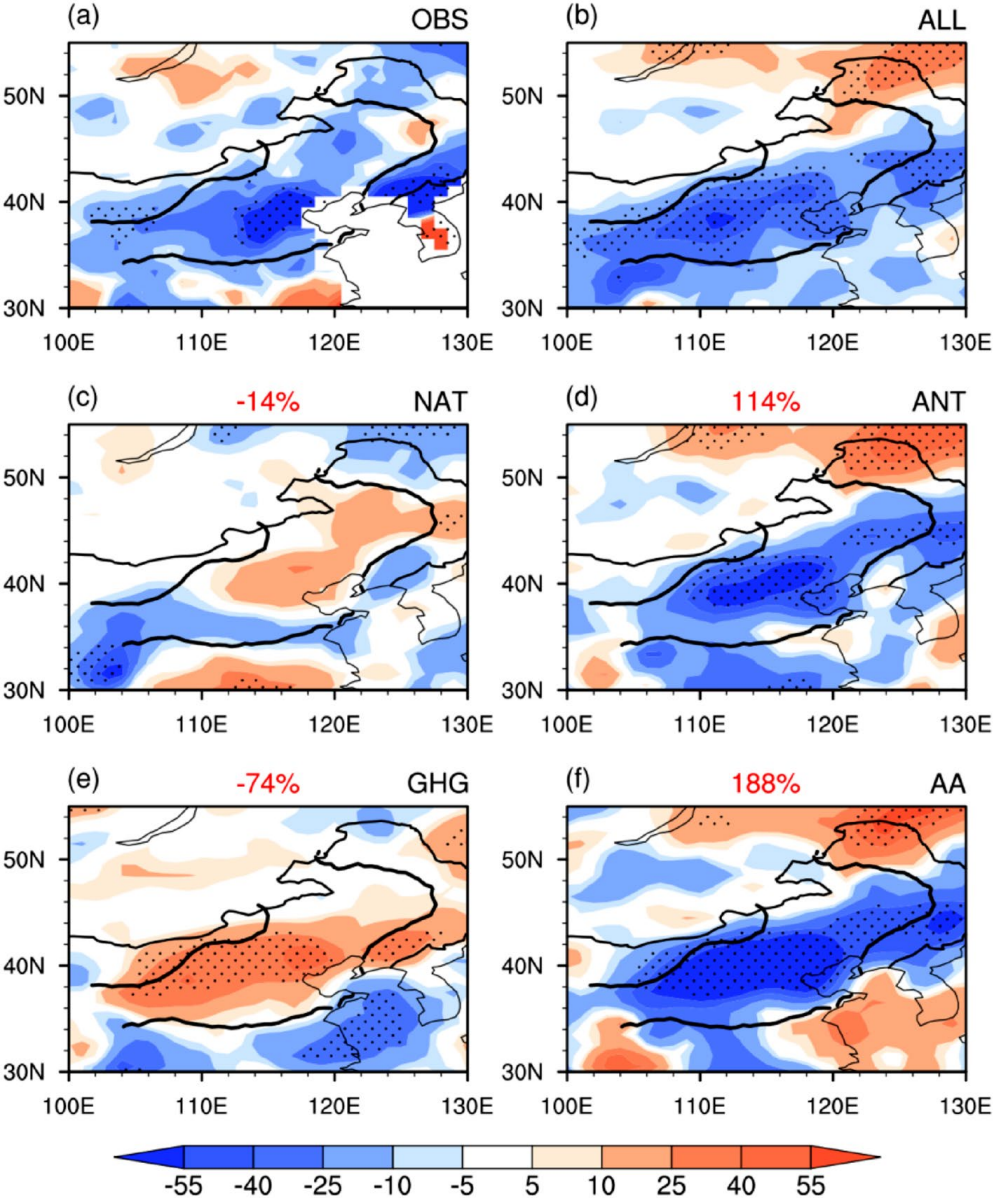
Linear trends of precipitation (unit: mm month^−1^ 55 yr^−1^) in August during 1951–2005. (**a**) Observational precipitation trend averaged between CRU TS version 4.01^21^ (URL: http://crudata.uea.ac.uk/cru/data/hrg/cru_ts_4.01/) and GPCC V7^22^ (URL: https://psl.noaa.gov/data/gridded/data.gpcc.html). The multimodel ensemble simulations of (**b**) historical run, (**c**) natural forcing run, (**d**) anthropogenic forcing run, (**e**) GHG forcing run, and (**f**) aerosol forcing run. Regions covered with dots feature linear trends significant at the 90% confidence level. The black curves indicate the boundary of the TCZ domain. The numbers at the center-top of the figures denote the relative contribution percentages of the corresponding external forcings. This Figure is created by the NCAR Command Language (version 6.4.0 & URL: http://www.ncl.ucar.edu/Download)^27^.

Before investigating the relative contributions of the external and natural forcings to the declining trend of August precipitation over the TCZ, we first evaluate the ability of the historical simulations from 32 CMIP5 models to reproduce the observed decreasing trend (Fig. 2). As shown in Fig. 2, only 3 (i.e., CCSM4, GFDL-CM3, and GISS-E2-R) of the 32 CMIP5 models can capture the observed decreasing trend. Therefore, the following analyses are based on the MME of these three models. Figure 3 displays the spatial distribution of the linear trends of August precipitation over the TCZ in the observation and individual external forcings. Here, the observation is defined as the average of CRU and GPCC. The observed significant decreasing trend can be well reproduced by all forcing run both in terms of the spatial distribution and the amplitude (Fig. 3a,b). The natural forcing produces an increasing precipitation trend over most parts of the TCZ but cannot pass the 90% confidence level (Fig. 3c). This suggests that natural forcing plays a limited role in the recent declining trend of TCZ precipitation in late summer. The anthropogenic forcing run shows a high similarity to the all forcing run (Fig. 3d). This implies that anthropogenic forcing is the dominant factor (Fig. 3d). The anthropogenic forcing is the sum of the GHG forcing and AA forcing^25^. It is found that the AA forcing produces a pronounced negative precipitation trend in the TCZ, with a spatial distribution similar to the all forcing runs (Fig. 3f). In contrast, the GHG forcing leads to an increasing precipitation trend over most of the TCZ, which is in sharp contrast to the observation (Fig. 3a,e). This suggests that the increasing GHGs could lead to a wetter condition over the TCZ. Hence, the above evidence indicates that the decreasing trend of late-summer precipitation over the TCZ over several recent decades is mainly attributed to AA forcing. The GHG forcing has a negative contribution. The contribution of natural forcing according to the MME is slightly negative indicating wetting but not statistically significant trend.

We further quantitatively estimate the relative contributions of different external forcings by calculating the relative contribution percentage (RCP). Here, the RCP is defined as the ratio of the precipitation trend in different forcing runs to the all forcing run (numbers in red at the center-top of Fig. 3c–f). As expected, the AA forcing is the dominant external forcing that contributes to the precipitation decrease over the TCZ, with an RCP of approximately 188%. The contribution of GHG forcing is negative, with an RCP of approximately − 74%. In comparison to natural forcing (RCP = − 14%), anthropogenic forcing plays a more important role (RCP = 114%). It should be noted that the RCP we derived here is estimated using linear theory proposed by Taylor et al.^25^, and has been widely used by other studies^29–31^. The effects of nonlinear interaction between the GHG forcing and AA forcing may exist in the real climate responses. However, the detailed investigation of the nonlinearity is beyond the scope of this study. To what extent will the nonlinearity among different external forcings influence the precipitation changes over the TCZ remains to be explored.

Studies have revealed that atmospheric circulation anomalies play a key role in modulating precipitation over the TCZ both on the interannual and interdecadal timescales^10,32–34^. Specifically, a stronger western North Pacific (WNP) subtropical high and the associated southerly wind anomalies over East Asia would result in above-normal precipitation over the TCZ by bringing more water vapor from southern oceans^28,32–34^. The reverse conditions are true for a weakened WNP subtropical high. Thus, in the following, atmospheric circulation is investigated to understand how these external forcings contribute to the precipitation trend over the TCZ during 1951–2005.

**Figure 4:**
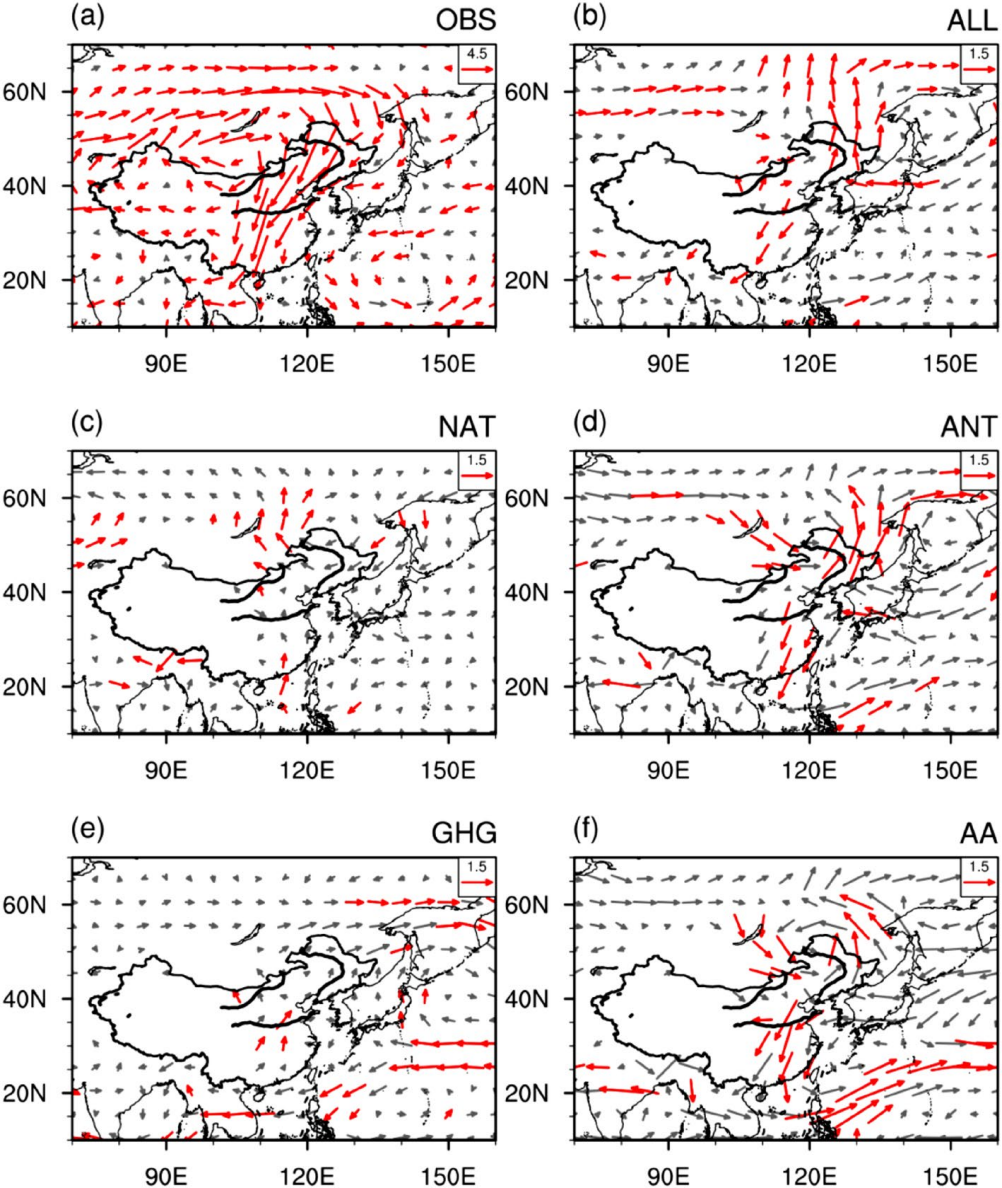
Linear trends of horizontal winds at 850 hPa (unit: m s^−1^ month^−1^ 55 yr^−1^). Observation in (**a**) is represented by the NCEP–NCAR reanalysis 1^23^ (URL: https://psl.noaa.gov/data/gridded/data.ncep.reanalysis.html). The multimodel ensemble simulations of (**b**) historical run, (**c**) natural forcing run, (**d**) anthropogenic forcing run, (**e**) GHG forcing run, and (**f**) aerosol forcing run. The arrows in red represent at least one direction of horizontal winds’ linear trend significant at the 90% confidence level. This Figure is created by the NCAR Command Language (version 6.4.0 & URL: http://www.ncl.ucar.edu/Download)^27^.

Figure 4 shows the linear trend of 850-hPa winds derived from observation and different forcings. In the observation, the TCZ is covered by pronounced anomalous northerly winds with a southward extension to southern China, which inhibits the northward transport of water vapor to the TCZ and explains the marked decreasing precipitation trend there (Figs. 3a and 4a). Note that the results shown in Fig. 4a derived from the NCEP–NCAR reanalysis 1 can be confirmed by other reanalysis datasets (e.g., CERA20c from ECWMF and 20CRv3 from NOAA), although amplitudes of the northerly wind trend over eastern China are slightly weaker in CERA20c and 20CRv3 (Please see Fig. S1 in the supporting information). The notable northerly wind trend can generally be reproduced by the all forcing run, though with a weaker magnitude (Fig. 4b). This is consistent with the fact that the all forcing run can reasonably capture the observed precipitation trend over the TCZ (Fig. 3b). The trend of 850-hPa winds in the natural forcing is weak and insignificant over most portions of East Asia and the WNP (Fig. 4c). Similar to the all forcing run, the prevailing northerly wind anomalies occupied eastern China in the anthropogenic forcing, which is mainly attributable to the AA forcing (Fig. 4f). In addition, westerly wind anomalies to the north of TCZ are also observed (Fig. 4c,f). The strengthened low-level northerly wind anomalies obstruct water vapor transport from the southern oceans to the TCZ regions. Meanwhile, the westerly wind anomalies to the north of TCZ generate divergence in the lower troposphere over the TCZ with the northerly wind anomalies to the south of TCZ, which further suppresses the TCZ precipitation. In general, the trend of atmosphere circulation in the lower troposphere can explain the declining trend of precipitation over the TCZ. In contrast, under the GHG forcing, significant southerly wind anomalies appear over eastern China, enhancing the water vapor supply towards the TCZ and therefore facilitating an increase in precipitation there.

**Figure 5:**
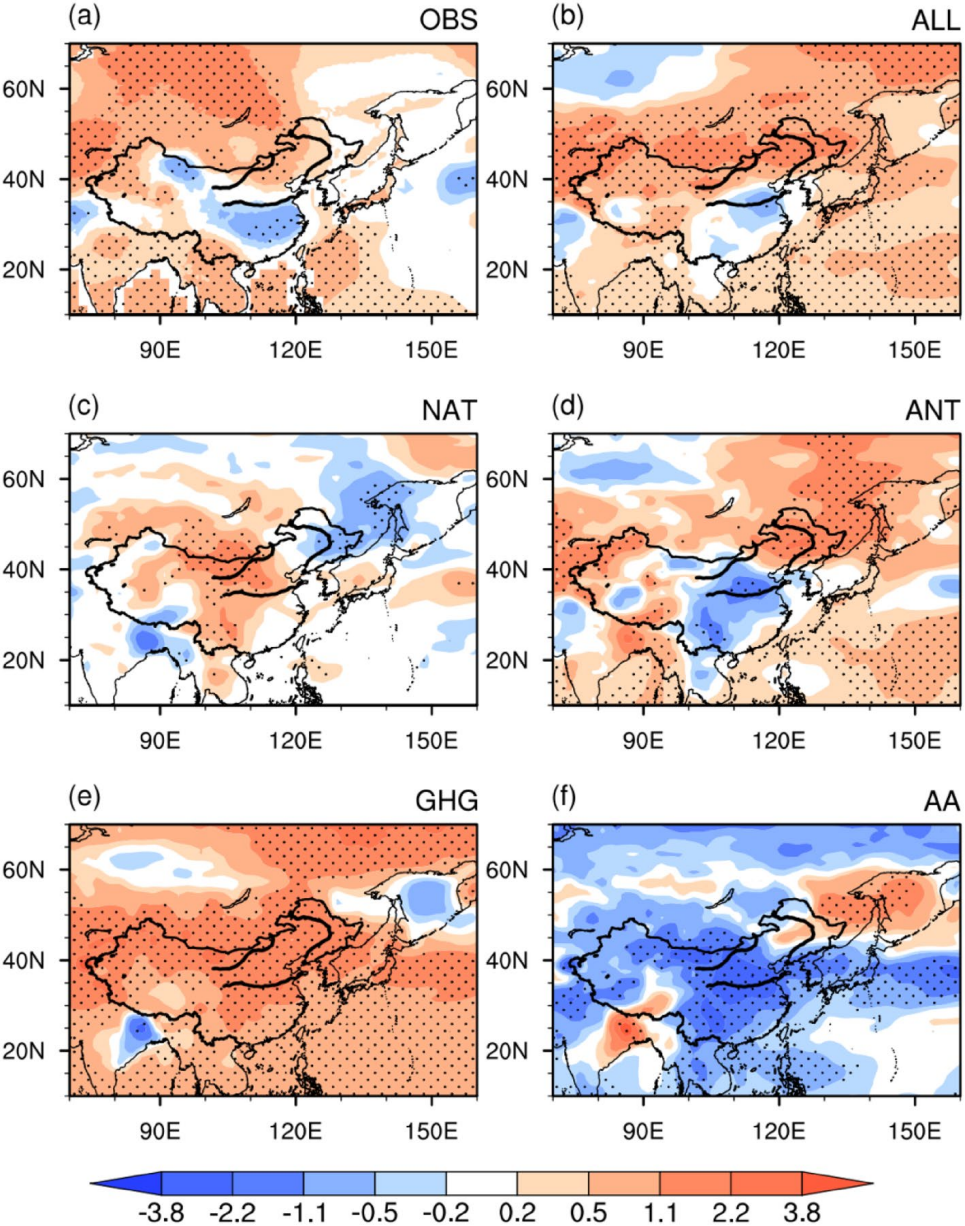
Linear trends of surface temperature (unit: °C month^−1^ 55 yr^−1^). The linear trend of surface air temperature (sea surface temperature) in (**a**) is derived from CRU TS version 4.01^21^ (URL: http://crudata.uea.ac.uk/cru/data/hrg/cru_ts_4.01/) (ERSST v3b^24^ (URL: https://psl.noaa.gov/data/gridded/data.noaa.ersst.v3.html)). The multimodel ensemble simulations of (**b**) historical run, (**c**) natural forcing run, (**d**) anthropogenic forcing run, (**e**) GHG forcing run, and (**f**) aerosol forcing run. Regions covered with dots feature linear trends significant at the 90% confidence level. This Figure is created by the NCAR Command Language (version 6.4.0 & URL: http://www.ncl.ucar.edu/Download)^27^.

To further explore the underlying process of how different external forcings alter the precipitation trend over the TCZ by modulating the atmospheric circulation, we examine linear trends in SAT and SST. In the observation, significant warming appears over the mid-high latitudes of Eurasia and the WNP, and prominent cooling occurs over the southeastern part of China (Fig. 5a). Due to the distinct heat capacities of the land and sea, the simultaneous appearance of SST warming in the WNP and SAT cooling over southern China may reduce the thermal contrast between the offshore continent of China and the WNP. This would decrease the East Asian summer monsoon, lead to northerly wind anomalies over East Asia (Fig. 4a) and result in a negative precipitation trend over the TCZ (Fig. 3a). The inverse change in the SAT over the WNP and southern part of China, which reduces the land-sea SAT gradient, can be captured by the all forcing run and the anthropogenic forcing run (Fig. 5b,d). In the natural forcing run, the surface temperature trends over the WNP and southern China are weak and insignificant (Fig. 5c). In the greenhouse forcing run, the SAT warming over the Eurasian continent is stronger than the SST warming in the WNP, leading to an increase in the land-sea thermal contrast (Fig. 5e), which would lead to southerly wind anomalies over East China and above-normal precipitation over the TCZ. In contrast, for the AA forcing, the SAT cooling over the Eurasian continent is larger than the SST cooling in the WNP due to the direct radiative effect, which results in the shrinking of the land-sea SAT gradient and in the presence of northerly wind anomalies over East Asia (Fig. 5f).

In summary, the weakening of the land-sea temperature gradient in the observation and several external forcing runs (i.e., all forcing run, anthropogenic forcing run, and AA forcing run) could induce northerly wind anomalies over eastern China (Fig. 4a,b,d,e). In these runs, the climatology southerly winds along the western fringe of the WNP subtropical high, which provide water vapor to the TCZ, would be weakened, and thus, precipitation over the TCZ is suppressed (Fig. 3a,b,d,e). In contrast to the AA forcing, the situation in the GHG forcing run favors an increase in precipitation over the TCZ.

## Summary and discussions

Based on the observation and 32 model simulations from CMIP5, the relative contributions of different forcings (i.e., all forcing, natural forcing, anthropogenic forcing, GHG forcing, and AA forcing) to the marked drying trend of the TCZ since the early 1950s are investigated. Observations show that precipitation over the TCZ in late summer (i.e., August) experienced a prominent declining trend during recent decades. A comparison of several external forcing runs suggests that the AA forcing plays a dominant role in the decreasing precipitation over the TCZ, with an RCP of 188%. The AA forcing can induce notable low-level northerly wind anomalies over eastern China, which inhibit water vapor transport towards the TCZ and suppress precipitation by weakening the land-sea thermal contrast between Eurasia and the surrounding oceans. A contribution in the opposite direction (with an RCP of − 74%) is found for the GHG forcing, offsetting the contribution of the AA forcing. As a combined effect of GHG forcing and AA forcing, anthropogenic forcing exerts a positive contribution with an RCP of 114%. The contribution of natural forcing is relatively weak because the atmospheric responses are not obvious.

It is worth noting that the AA forcing can not only affect large-scale precipitation by modulating the atmospheric circulation but can also alter the local precipitation via microphysical cloud processes, as indicated by previous studies^35–37^. For example, Qian et al.^35^ reported that an increase in the AA concentration would increase the droplet number concentration and reduce the size of cloud droplets, postponing the formation of raindrops; therefore, the frequency and amount of light rain over northeastern China would be significantly decreased. According to the public record, the air conditions of megacities over the TCZ, such as Beijing, Tianjing, Shijiazhuang, and others, have experienced serious deterioration because of rapid industrialization. Therefore, we speculate that the increase in local AA emissions may be partly responsible for the precipitation decrease over the TCZ. This issue will be further explored in our future studies.

This study shows that 3 out of the 32 CMIP5 models can capture the observed decreasing trend of the late-summer TCZ rainfall. It remains unclear whether the ability of the models in reproducing the observed precipitation trend in late summer over the TCZ is attributed to SST biases in the models. To address this issue, we have examined spatial distribution of the biases in climatological SST in August over 1951–2005 in 32 CMIP5 models relative to the observation (Please see Fig. S2 in the supporting information). As shown in Fig. S2, SST cooling biases in central North Pacific and North Atlantic and SST warming biases in eastern coast of South and North America can be observed in most CMIP5 models. In general, spatial patterns of SST biases in 32 CMIP5 models present some common features. In addition, we have calculated the spatial correlation coefficients of global SST biases between CCSM4, GFDL-CM3, GISS-E2-R and other CMIP5 models (Please see Fig. S3 in the supporting information). From Fig. S3, SST biases in many models bears high similarity to those in CCSM4, GFDL-CM3, and GISS-E2-R with large spatial correlation coefficients exceeding 0.8. This indicates that the climatological SST biases may not be the dominant source of biases in simulating the TCZ precipitation trend in late summer. At present, it is still unclear to us why only 3 out of 32 CMIP5 models can reproduce the significant TCZ precipitation declining in late summer over 1951–2005. The capability of coupled-models in simulating the regional precipitation may be vary greatly due to the parametric schemes and other factors. Previous studies have demonstrated that late-summer precipitation in TCZ can be modulated by many factors including the tropical SST, East Asia summer monsoon, and atmospheric circulation in mid-high latitude^32–34,38,39^. The incapability of the models in simulating these factors may influence the performance of the models in reproducing the TCZ precipitation declining in August. The factors responsible for the ability of the models in simulating the long-term trend of late-summer precipitation over TCZ should be further investigated.

## Supplementary Information

Supplementary Information
