# Supplementary material for: Roles of anthropogenic forcings in the observed trend of decreasing late-summer precipitation over the East Asian transitional climate zone
Source: Sci Rep. 2021 Mar 2;11:4935. doi: 10.1038/s41598-021-84470-9 (PMC7925582; doi:10.1038/s41598-021-84470-9)
Supplement: Supplementary file 1 — Supplementary Information [file 41598_2021_84470_MOESM1_ESM.docx]

**Supplementary Information**

**Roles of anthropogenic forcings in the observed trend of decreasing late-summer precipitation over the East Asian transitional climate zone**

Wei Zhao, Wen Chen, Shangfeng Chen, Hainan Gong, and Tianjiao Ma

**Table S1** Information on the CMIP5 models employed in this study.

| **Model name** | **Source** | **Horizontal Resolution** |
| --- | --- | --- |
| ACCESS1-0 | CSIRO (Commonwealth Scientific and Industrial Research Organisation, Australia), and BOM (Bureau of Meteorology, Australia) | 145×192 |
| ACCESS1-3 | CSIRO (Commonwealth Scientific and Industrial Research Organisation, Australia), and BOM (Bureau of Meteorology, Australia) | 145×192 |
| bcc-csm1-1 | Beijing Climate Center, China Meteorological Administration, China | 64×128 |
| BNU-ESM | Beijing Normal University, China | 64×128 |
| CanESM2 | Canadian Centre for Climate Modelling and Analysis, Canada | 64×128 |
| CCSM4 | National Center for Atmospheric Research (NCAR), USA | 192×288 |
| CESM1-BGC | National Science Foundation, Department of Energy, National Center for Atmospheric Research | 192×288 |
| CESM1-CAM5 | National Science Foundation, Department of Energy, National Center for Atmospheric Research | 192×288 |
| CSIRO-Mk3-6-0 | Australian Commonwealth Scientific and Industrial Research Organization, Australia | 96×192 |
| CMCC-CM | Centro Euro-Mediterraneo per I Cambiamenti Climatici | 240×480 |
| CMCC-CMS | Centro Euro-Mediterraneo per I Cambiamenti Climatici | 96×192 |
| CNRM-CM5 | Centre National de Recherches Meteorologiques, Meteo-France, France | 128×256 |
| FGOALS-s2 | Institute of Atmospheric Physics, Chinese Academy of Sciences, China | 60×128 |
| FIO-ESM | The First Institute of Oceanography, SOA, China | 64×128 |
| GFDL-CM3 | Geophysical Fluid Dynamics Laboratory, USA | 90×144 |
| GFDL-ESM2G | Geophysical Fluid Dynamics Laboratory, USA | 90×144 |
| GFDL-ESM2M | Geophysical Fluid Dynamics Laboratory, USA | 90×144 |
| GISS-E2-H | NASA Goddard Institute for Space Studies, USA | 90×144 |
| GISS-E2-R | NASA Goddard Institute for Space Studies, USA | 90×144 |
| GISS-E2-R-CC | NASA Goddard Institute for Space Studies, USA | 90×144 |
| HadGEM2-AO | Met Office Hadley Centre, United Kingdom | 145×192 |
| inmcm4 | Institute for Numerical Mathematics | 120×180 |
| IPSL-CM5A-LR | Institut Pierre-Simon Laplace, France | 96×96 |
| IPSL-CM5A-MR | Institut Pierre-Simon Laplace, France | 96×96 |
| IPSL-CM5B-LR | Institut Pierre-Simon Laplace, France | 96×96 |
| MIROC5 | AORI, NIES, JAMSTEC, Japan | 128×256 |
| MIROC-ESM | AORI, NIES, JAMSTEC, Japan | 64×128 |
| MIROC-ESM-CHEM | AORI, NIES, JAMSTEC, Japan | 64×128 |
| MPI-ESM-LR | Max Planck Institute for Meteorology, Germany | 96×192 |
| MPI-ESM-MR | Max Planck Institute for Meteorology, Germany | 96×192 |
| MRI-CGCM3 | Meteorological Research Institute, Japan | 160×320 |
| NorESM1-M | Norwegian Climate Centre, Norway | 96×144 |


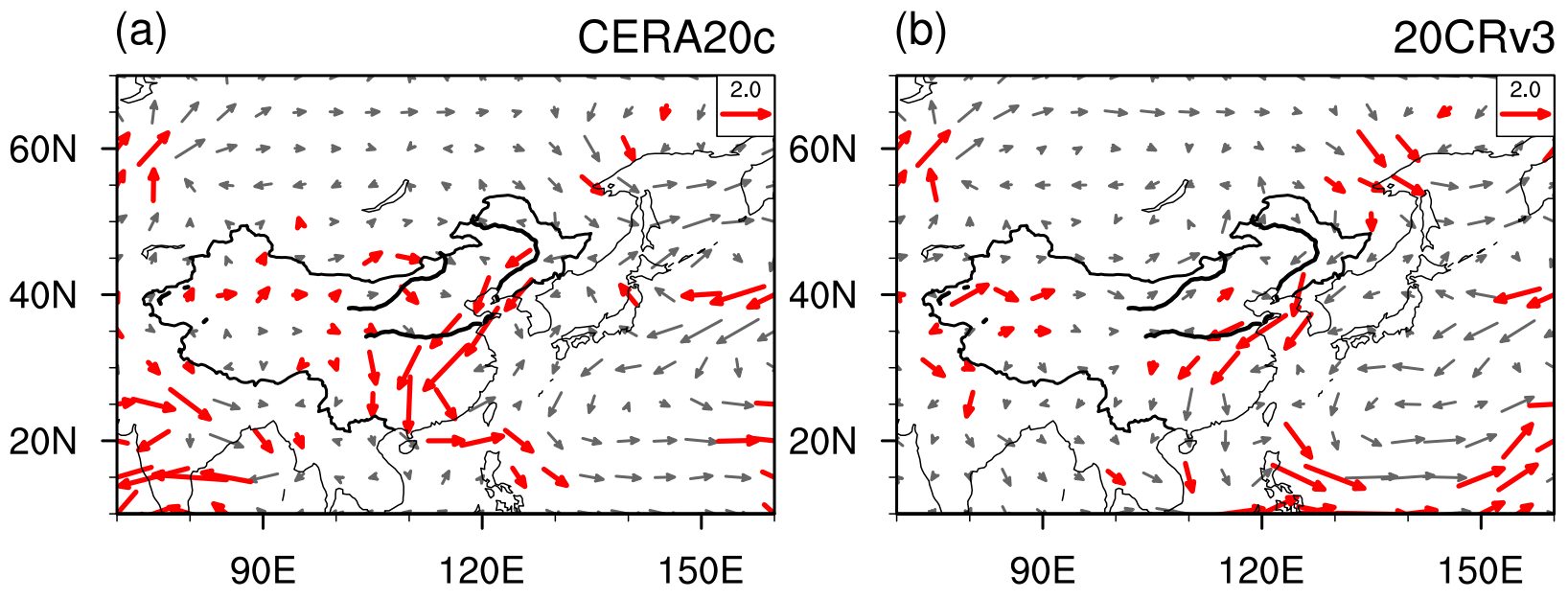


**Figure S1.** Linear trends of horizontal winds at 850hPa (unit: m s^-1^ month^-1^ 55 yr^-1^) derived from (a) CERA20c (URL: https://www.ecmwf.int/en/forecasts/datasets/reanalysis-datasets/cera-20c) and (b) 20CRv3 (URL: https://psl.noaa.gov/data/gridded/data.20thC_ReanV3.html). The arrows in red represent at least one direction of horizontal winds’ linear trend significant at the 90% confidence level. This Figure is created by the NCAR Command Language (version 6.4.0 & URL: <http://www.ncl.ucar.edu/>Download) ^39^.


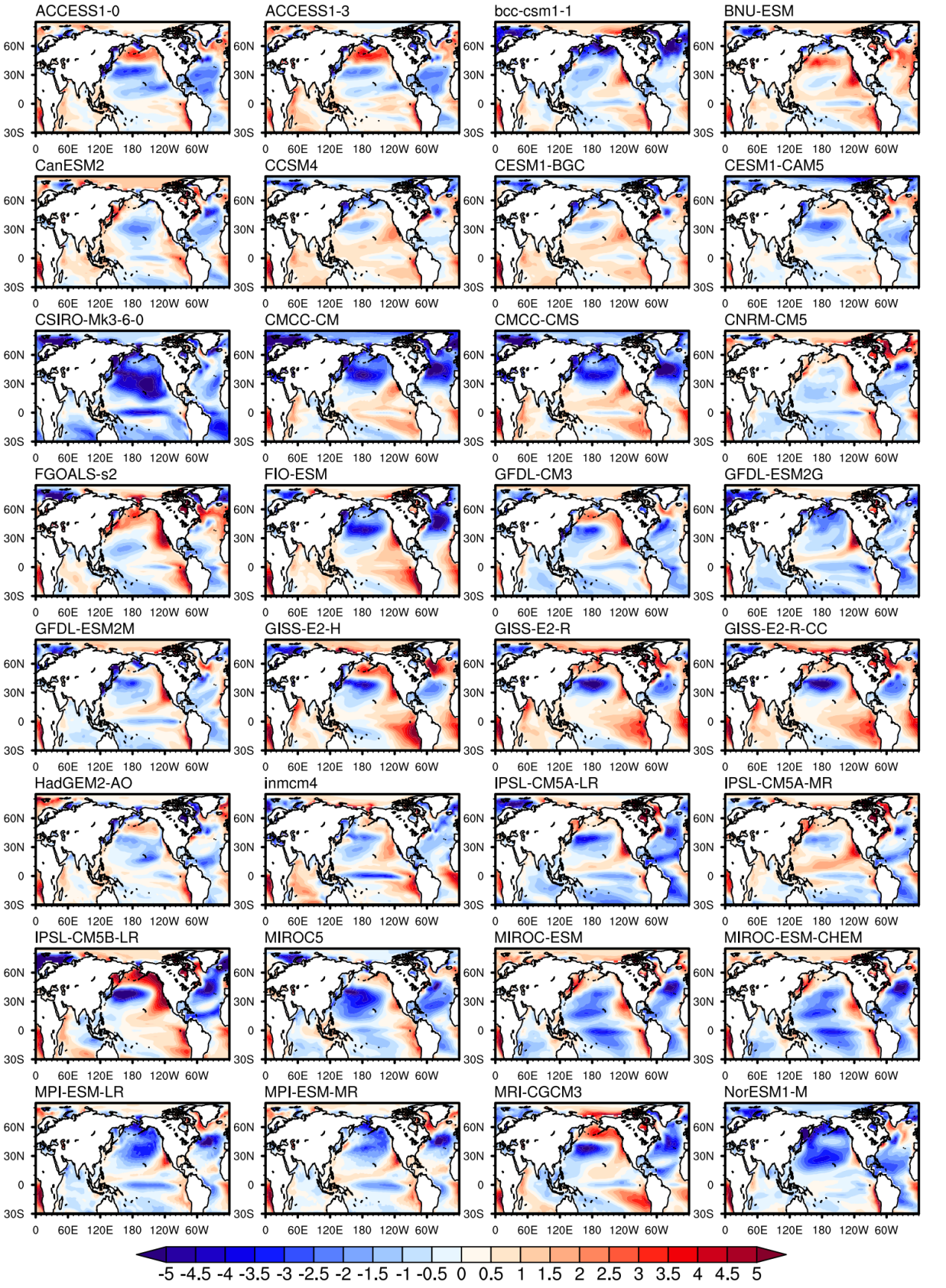


**Figure S2.** Differences of climatological sea surface temperature (SST; unit: ^o^C) in August during 1951-2005 simulated by 32 CMIP5 models relative to the observations. This Figure is created by the NCAR Command Language (version 6.4.0 & URL: <http://www.ncl.ucar.edu/>Download)^39^.


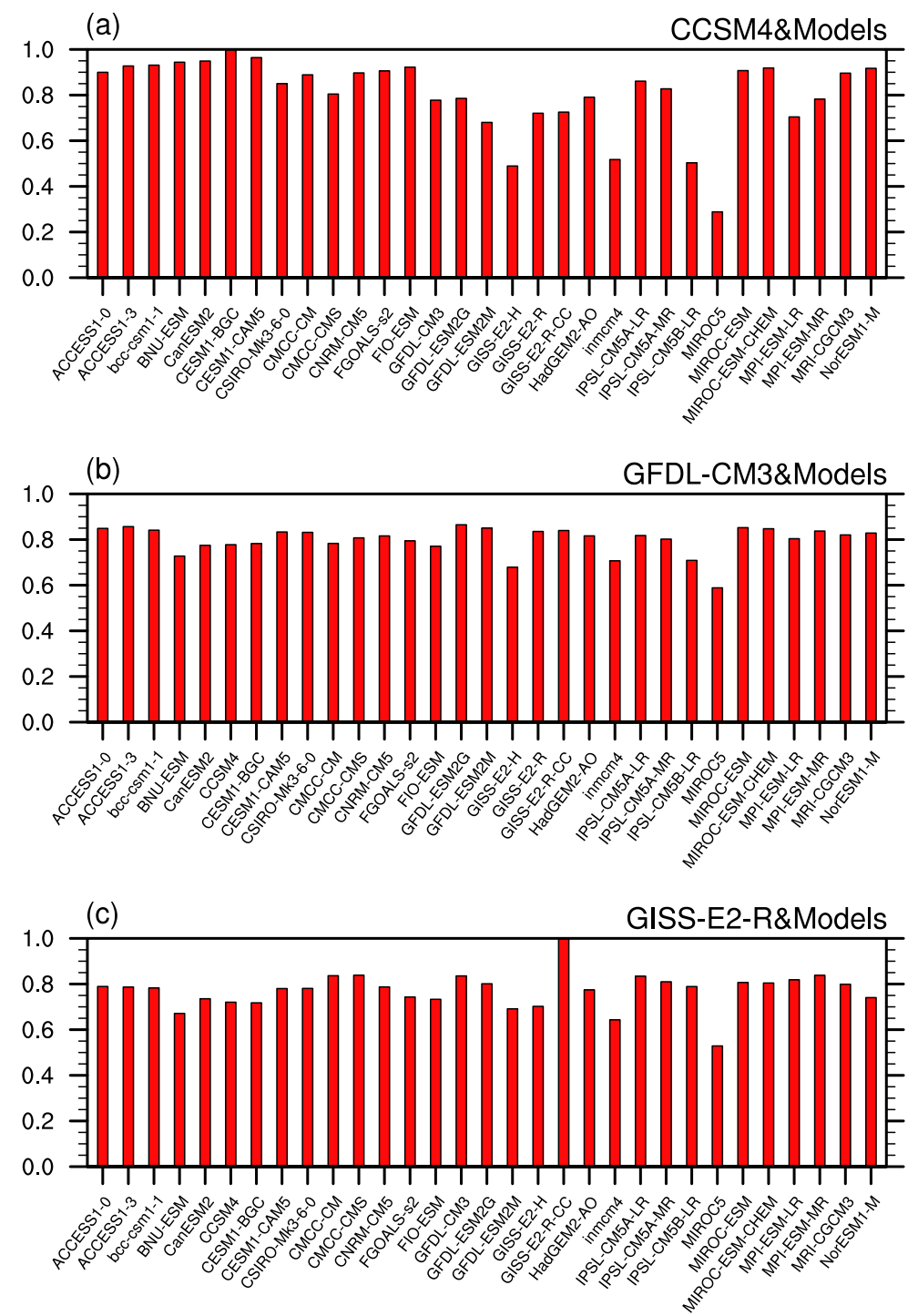


**Figure S3.** Spatial correlation coefficients between climatological SST biases of (a) CCSM4, (b) GFDL-CM3, (c) GISS-E2-R with other CMIP5 models over global region in August during 1951-2005. This Figure is created by the NCAR Command Language (version 6.4.0 & URL: <http://www.ncl.ucar.edu/>Download)^39^.
